# Splintered Fracture of Patella

**Published:** 1915-08

**Authors:** 


**Splintered Fracture of Patella.** Maurice Peraire, Bull. et Mem. de la Soc. de Med. de Paris, May 23, reports a case in a soldier aged 25, struck with a shrapnel bullet, left on the field of battle for 36 hours without irrigation so that suppuration occurred. Silver wire sutures were employed, in the open suppurating wound, both bone and ettendons being united. Antiseptic dressings and massage were also employed and union occurred by first intention (sic). The result was excellent.

